# Empowering clinical research in a decentralized world

**DOI:** 10.1038/s41746-021-00473-w

**Published:** 2021-07-01

**Authors:** Walter De Brouwer, Chirag J. Patel, Arjun K. Manrai, Isaac R. Rodriguez-Chavez, Nirav R. Shah

**Affiliations:** 1 Sharecare, Palo Alto, CA USA; 2 Clinical Excellence Research Center, Department of Medicine, Stanford University, Palo Alto, CA USA; 3 XY.Health, Inc., Cambridge, MA USA; 4 PRA Health Sciences, Charlottesville, VA USA

**Keywords:** Clinical trial design, Biomarkers

## Abstract

The COVID-19 pandemic has been a catalyst for the implementation of decentralized clinical trials (DCTs) enabled by digital health technologies (DHTs) in the field while curtailing in-person interactions and putting significant demands on health care resources. DHTs offer improvements in real-time data acquisition remotely while maintaining privacy and security. Here, we describe the implications of technologies, including edge computing, zero-trust environments, and federated computing in DCTs enabled by DHTs. Taken together, these technologies—in the setting of policy and regulation that enable their use while protecting the users—extend the scope and accelerate the pace of clinical research.

## Randomized control trials: an analog tool in a digital world

The gold standard of clinical research has been the randomized controlled trial (RCT). RCTs remain the most important approach to establish causality, addressing challenges of confounding and bias. However, only incremental improvements have been made since experiments were first conducted in medical research^1^. The coronavirus disease of 2019 (COVID-19), or the pandemic, by rapidly shifting clinical trials from analog to hybrid or full decentralized clinical trials (DCTs), has catalyzed the development and implementation of digital health technologies (DHTs) to support clinical research. This article describes the technologies that are modernizing clinical research, including DCTs.

RCTs were optimized for an “analog” world. Specifically, DHTs offer opportunities to advance clinical research along the axes of data acquisition or how data are collected and processed. Currently, data are processed in many instances via manual paper workflows, and new approaches for measuring digital endpoints and processing data on a trial participant’s device broadens possibilities for acquiring data more frequently or even continuously. Moreover, DHTs may enhance trial participants’ privacy to an extent not possible with manual workflows. Modern DCTs enabled by DHTs may enhance trial participant engagement through more active and secure bidirectional communication, leveraging digital technologies. Inan and colleagues^2^ lay a framework for digitalizing clinical trials that is extended in this paper. Specifically, we instantiate one version of DCTs enabled by DHTs and differentiate our work by describing privacy-preserving or “zero-trust” approaches to collect clinical data from digital endpoints to measure the effect of an intervention in situ via federated learning (FL).

## The new vision and methodology: bridging the efficacy–effectiveness gap

Historically, clinical trials have been designed with a site investigator-centric approach. Trial participants traveled to academic health centers where investigators and diagnostic technologies were concentrated in brick-and-mortar clinical trial sites on episodic schedules dictated by operational convenience rather than by disease natural history. The centralized trial approach, confined to unrealistic settings, generates theoretically unbiased findings (i.e., laboratory efficacy)^3^. However, beyond this setting, trial participants with multiple co-morbidities and imperfect adherence live in highly heterogeneous environments. Unsurprisingly, the “unbiased” findings of clinical trials do not always translate into real-world effectiveness^4^.

**Fig. 1:**
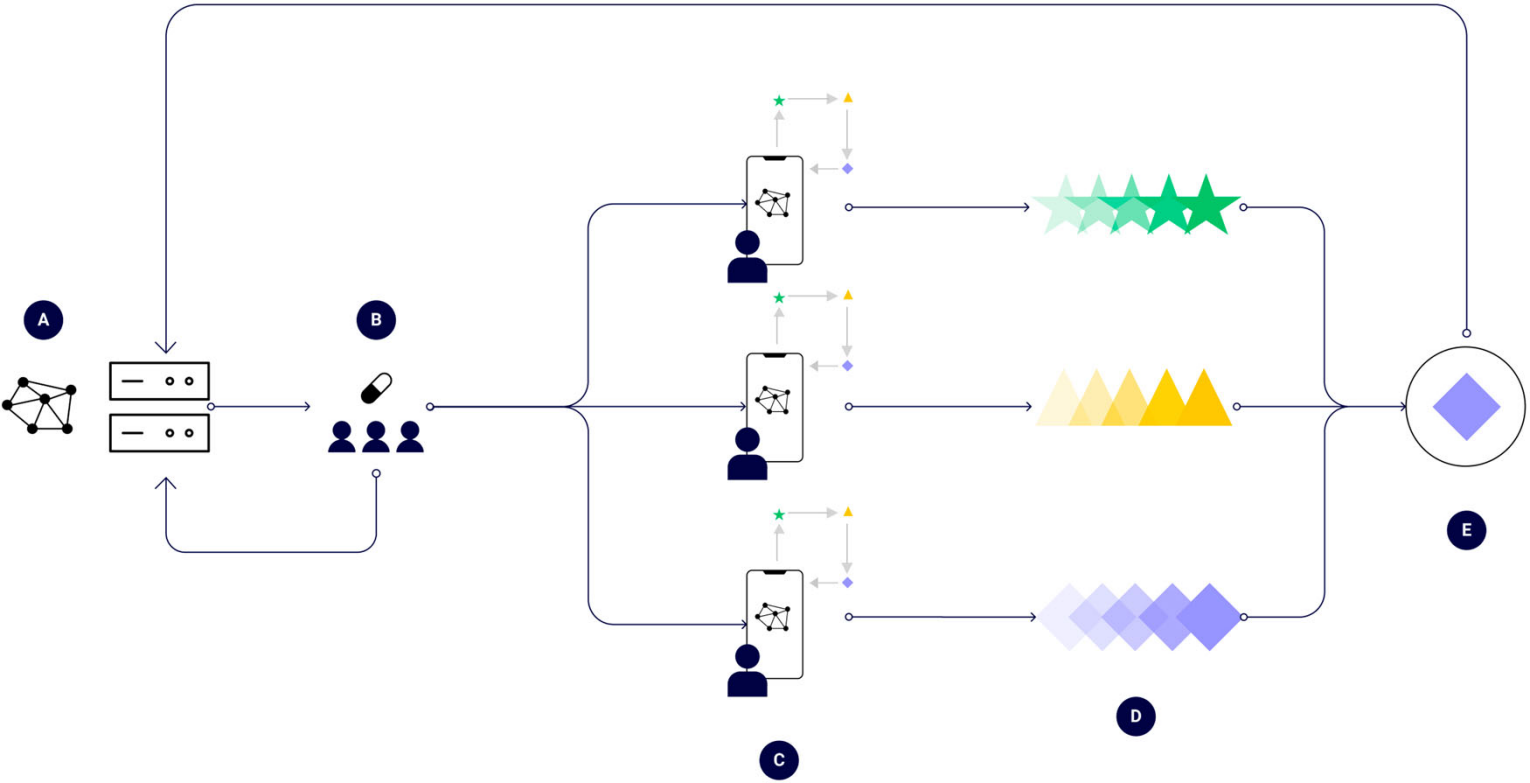
Process diagram for the decentralized execution of federated clinical trials. **A** A protocol is created and is digitally disseminated to consenting participants-to-be in a DCT, **B** Participants in a DCT are randomly exposed to a medical intervention, **C** Participants process their own data (e.g., digital biomarkers), **D** Findings are aggregated from each participant, and **E** The overall effect of the intervention is estimated as overall estimate, aggregated, and sent back.

New technologies for trial participants and remote data acquisition, processing, and analysis are increasingly available^5^. These DHTs are catalyzing the transition from centralized to remote settings in DCTs, enabling a remote-first paradigm in modern clinical trials. This paradigm offers a rapid evolution of clinical research in the coming years (Fig. 1).

Yet how to implement modern clinical trials such as DCTs using DHTs remains uncertain. Numerous considerations exist, including the selection of parameters for trial design, approval from institutional review boards, monitoring of trial participant’s safety and adherence, and the analysis of the data blinded to the trial team. As with traditional clinical trials, the privacy of trial participants and security of their data must be ensured.

### The implications of using emerging technologies on trial participant’s privacy and security

DHTs (e.g., smart devices, new wearables, and environmental sensors) are rapidly evolving. Already they play a significant role in enabling trial participants’ experience in modern clinical trials^6^. These technologies facilitate multiple trial-related activities: communication, enrollment, recruitment, consent, and continuous data acquisition. The validated capabilities to record novel digital endpoints have also been key to their use^7^. However, these new technologies may serve to provide new avenues to violate trust. “Zero-trust architectures”^8,9^ or networks assume that none of the DHTs used outside an organization’s network (in this case, the DHT network) can be trusted and require mutual authorization from the DHT client and server devices (Table 1).

**Table 1:** Glossary of key technical concepts.

Term	Definition	Implications
Digital biomarker	Biomarkers are defined characteristics that are measured as indicators of health, disease, or a response to an exposure or intervention, including therapeutic interventions and can help diagnose a disease, or predict future disease severity or outcomes, like measurements of blood pressure as an indicator of cardiovascular risks or measurements of blood sugar in diabetes. Biomarkers also are used to identify the best treatment for a patient, to monitor the safety of a therapy, or to find out whether a treatment is having the desired effect on the body. Digital refers to the method of collecting information: objective, quantifiable physiological and behavioral data are collected and measured by means of digital devices, such as portables, wearables, implantables, or digestibles	Compared to traditional biomarkers, digital biomarkers rely less often on human operators to collect data and, depending on the use case, may enhance self-collection (e.g., Patient Reported Outcome Measures), which can diversify the types of monitoring
Digital endpoints	Digital measurements of biomarkers that capture disease state from device data, such as sensors (image or audio based or biochemical assay)	Continuous collection of clinical endpoints possible to enhance monitoring and power
Digital health technologies	Digital health technologies refer to the use computing platforms, connectivity, software, and sensors for health care and related uses. These technologies span a wide range of uses, from applications in general wellness to applications as a medical device. They include technologies intended for use as a medical product, in a medical product, as companion diagnostics, or as an adjunct to other medical products (devices, drugs, and biologics). They may also be used to develop or study medical products	DHTs enable the collection of clinical data remotely, frequently, or continuously outside the brick-and-mortar clinical trial site and while keeping trial participants at their homes or in the ecosystems where they live
Digital twins	A computational approach, such as propensity score matching, that matches pairs of individuals exposed and unexposed to an intervention when a random allocation is not possible	Reduces experimental biases by correcting for confounding, mitigating the chances that a difference in outcomes observed during the trial is attributable to non-intervention factors
Edge computing	Machine learning architectures, tools, and approaches capable of performing on-device analytics for sensing and/or biomarker measurement	Removes need to transfer on-device data to central servers for data processing, enhancing data security, and participant anonymity
Federated learning (FL)	A computer engineering approach enabling multiple parties to re-train a shared model without sharing their data: an FL model processes and aggregates data from a private device without the data leaving the device	Removes need to transfer on-device data to central servers for model training to enhance security
Real-world data	According to the FDA^a^, real-world data are the data relating to patient health status and/or the delivery of health care routinely collected from electronic health records (EHRs), insurance claims, disease registries, and mobile devices	Increases efficiency of collection of clinical endpoints and predictor variables from administrative sources
Zero trust	A security trust framework that insists on verifying everything that needs to connect to corporate resources before granting access	Improves the safety of patient data in a significant way by conducting internal security protocols on devices and servers beyond peripheral ones alone

^a^https://www.fda.gov/science-research/science-and-research-special-topics/real-world-evidence.

Integrating clinical data obtained from DCTs (i.e., real-world settings) may add additional context between remote and in-person clinical interactions. Further, use of federated computing (FC) and zero trust approaches ensure trial participants’ privacy and deployment of complex digital endpoint-based machine learning (ML) algorithms while enabling classic double-blinded trial designs. The following section discusses the opportunities and challenges associated with these technological advances.

### Remote capture of high-throughput biomarkers and endpoints

Per U.S. Food and Drug Administration, “a biomarker is a piece of anatomical, physiological, or biochemical data that is used to diagnose or develop a treatment plan for a patient. A digital biomarker is simply a biomarker that is developed from data that are analyzed using advanced analytics and artificial intelligence (AI) to extract previously invisible insights.”^10^. An example of a biomarker is blood sugar which if detected with an electronic glucometer is a digital biomarker. In contrast, digital endpoints are measurements that capture an observable physiological “characteristic” of a participant through a DHT (e.g., an image or a signal). A standard or novel digital endpoint measures a biomarker that has clinical relevance. Examples of digital endpoints include the appearance, heat, or pallor of skin. Examples of standard digital endpoints are measurements for blood pressure or heart rate. Examples of novel digital endpoints include measurements for composite endpoints, such as the synergistic combination of heart rate and movement intensity^11^.

To determine the efficacy and safety of an investigational medical product, novel digital endpoints^12^ may complement standard endpoints, both because of their predictive capabilities and their feasibility of inclusion in DCTs^13^.

New techniques from ML may play a critical role in deriving utility from information-dense digital endpoints gathered from DHTs. Complex digital signals such as images that allow for the derivation of new clinical endpoints (such as for diabetic retinopathy^14^ or cancerous skin lesions^15^) have shown promise, reaching clinician-grade quality in multiple novel trials.

Other DHTs capable of digital endpoint monitoring include low-risk, consumer-grade devices that may measure clinically relevant endpoints, such as blood oxygen saturation, pulse, respiratory rate, and temperature. These technologies may also engage signal processing and ML algorithms that operate either on- or off-device (i.e., in the cloud and separated from the signal-capturing DHT)^11,16^.

### The environmental and contextual sensory capabilities of DHTs may support novel trial designs

A well-designed clinical trial should characterize the efficacy of an intervention after accounting for environmental and social determinants. However, traditional clinical research methods rarely account for these determinants. In contrast, DHTs often have sensors capable of automatically collecting location-based contextual factors related to participants, giving investigators additional visibility into the underlying determinants of observed endpoints.

Examples of such factors—collectively, the “exposome”—include air pollution (such as ozone or carbon monoxide) or particulate matter (such as pollen or nanometric plastics)^17^. Modalities such as satellite imaging may render additional insights, such as characteristics of the built environment, including access to green spaces for physical activity, and quality of food and water sources^18^. Digital tracking of the exposome may enhance the generalizability of clinical research findings into real-world settings.

### Data-intensive challenges exist for transforming digital biomarkers into digital endpoints

There are significant challenges associated with characterizing digital biomarkers (Table 1) and transforming them into validated digital endpoints in novel trial designs. First, digital biomarkers must be accurate indicators of disease status reliably measured as digital endpoints based on rigorous good clinical practice standards: reliably and accurately indicating changes in disease onset, progression, or improvement.

Additionally, there may be challenges in participants’ recruitment and adherence to investigational medical interventions captured using DHTs^19–21^. Clinical investigations have faced issues ensuring adherence of remote trial participants to an experimental treatment regimen. Existing strategies, such as automated electronic reminders, may help mitigate non-adherence and reinforce the viability of digital endpoints^22,23^.

## Federated computation and zero-trust techniques for DCTs

A major advantage of DCTs is the availability of numerous digital biomarkers and digital endpoints that can be measured remotely and continuously. These are in turn useful to derive novel digital (composite) endpoints with improved predictive potential. A second advantage is the ability to monitor adverse events in real time directly through linked electronic health records. A third advantage includes reduced costs of clinical assessments during clinical trial follow-up, with numerous digital endpoints collected continuously via DHTs.

### The promise of federated computation and zero-trust platforms for DCTs

Advances in FL (Table 1) may ensure trial participants’ privacy while enabling computation-intensive administration of DCTs, including the need to continuously monitor digital endpoints, perform linkage of remote data, and apply ML on digital endpoints^24^.

We present a conceptual schematic that demonstrates the process (Fig. 1). First, a “master” compute node becomes responsible for administering the trial, encoding the logic behind the trial design (Fig. 1A). This master node transmits a baseline model to trial participant nodes. The intervention is deployed randomly or prospectively (Fig. 1B). Next, individual client processing trial participant data, such as generating a digital endpoint (Fig. 1C) to input into a FL algorithm, which learns an output for a trial participant (Fig. 1D) while optimizing the overall population-level effect of the intervention (Fig. 1E). Specifically, the federated learner is optimizing overall the trial participant nodes securely (no data leaves the device). The learnings are aggregated and sent back to the master compute node. Zero-trust authentication (Table 1) occurs among all respective nodes of the network. One emerging technology to achieve zero-trust architectures in health care is via blockchain-based technologies^25^ and this has been evaluated in medical image sharing^26^.

### Ensuring trial participant privacy

The most significant advance that FC brings to DCTs is the groundwork to enable computationally intensive encryption for trial participants’ privacy.

Federated edge computing enables medical data portability (Table 1). Designs derived from edge models could then be used for powering key use cases such as improving cost of care models or for proactive risk management for a variety of end users—including payors, pharmaceutical companies, providers, and clinical research organizations. For example, pharmaceutical companies could use horizontal federation to introduce synthetic control arms, while providers could use vertical federation to combine medical data clouds between sources of clinical care to advance biomedical science and improve trial participant care.

### Potential limitations of DCTs enabled by DHTs

We anticipate three near- to longer-term limitations of DCTs enabled by DHTs. First, while participant recruitment may be potentially more accessible and cheaper via DCTs, trial participant retention could be a major challenge. Incentivizing trial participant engagement via ownership requires validation. Second, many primary outcomes of current trials will need to be newly designed for DCTs, and further still, many primary outcomes may not have a DCT analog. Third, DCT-related digital endpoints and infrastructure will need extensive verification and validation along with big data-processing capabilities. Fourth, clinical data standards for portability and interchangeability from multiple e-sources should be implemented to ensure data integrity and quality and to ensure widespread adoption.

These limitations might be addressed by four approaches in the coming years. First, we need more sophistication, refinement, and variety of DHTs to measure digital endpoints remotely. Second, we require a stronger emphasis on the verification, validation, justification, and usability of DHTs (i.e., wearables) to ensure data quality, integrity, interchangeability, and portability obtained from measuring digital endpoints. Third, health care and clinical research policies need to evolve simultaneously across multiple jurisdictions and countries to support the broad inclusion and recognition of DHTs and digital endpoints in all therapeutic areas by payers and regulatory agencies. Fourth, we must foster improvements in real-time data analysis by integrating outputs of digital endpoints and clinical data derived from multiple e-sources with AI/ML. This will allow a more comprehensive and holistic understanding of the health status of individual patients and participants in DCTs. Collectively, these actions can help us achieve the vision of health research advanced by DCTs enabled by DHTs.

## Conclusion

Traditional trials are costly^27,28^, laborious, and time-consuming^29^, often evaluating investigational medical products under stringent conditions. Moreover, during COVID-19, the operational feasibility of these trials has been challenged, predicting irreversible changes after the pandemic.

In contrast, DCTs enabled by DHTs exploit heterogenous and continuous data collection in real-world settings, allowing investigators to better evaluate disease status relative to a novel medical intervention and while determining the safety and efficacy of an investigational medical product. These trials may use technologies such as FL and edge computing to analyze data, advance clinical research, and enhance trial participants’ welfare.
